# Drug Resistance Assessment of Commonly Used Anthelmintics, and Their Combination Effect in Naturally Infected Sheep: The Case of Kaffa Zone, Southwestern Ethiopia

**DOI:** 10.1002/vms3.71147

**Published:** 2026-08-05

**Authors:** Nahom Belay, Asrat Arke, Lamrot Tesfaye

**Affiliations:** ^1^ Southwestern Ethiopia Agricultural Research Institute, Bonga Agricultural Research Center Bonga Ethiopia

**Keywords:** anthelmintic, combination, efficacy, Kaffa, resistance, sheep

## Abstract

**Background:**

Gastrointestinal nematode (GIN) infections are widely distributed across the country and are responsible for considerable losses in small ruminant production and the emergence of anthelmintic resistance has intensified the challenge. This study evaluated the efficacy of widely used commercial anthelmintics and examined whether combining different drug classes enhances their effectiveness in naturally infected sheep in Kaffa Zone.

**Materials and Methods:**

An experimental trial was conducted using 275 sheep that shed at least 150 eggs per gram count, randomly divided into six groups. Faecal samples were collected from each animal before and after treatment using selected anthelmintics, and the faecal egg count reduction test was employed to identify resistance towards each drug.

**Results:**

Faecal egg count reduction test values and corresponding lower 95% confidence limit were 90.31% and 88.51% for albendazole, 91.39% and 89.53% for tetramisole, and 95.84% and 95.05% for ivermectin, respectively. The findings indicated reduced effectiveness of albendazole and tetramisole, while ivermectin maintained acceptable efficacy. Combination treatments showed markedly higher reductions—99.11% for albendazole with ivermectin and 99.27% for tetramisole with ivermectin. The range of faecal egg counts of infected sheep was from 350 to 6750 EPG before treatment and from 0 to 1550 EPG after treatment. The overall mean of faecal egg counts of infected sheep was 1881.5 ± 69.7 (95% CI: 1744.2–2018.9) and 229.9 ± 28.4 (95% CI: 174.1–285.8) pre‐treatment and post‐treatment, respectively.

**Conclusion:**

The findings suggest the presence of emerging anthelmintic resistance to albendazole and tetramisole in the study area and highlight the potential usefulness of combination anthelmintic strategies. Continued monitoring and integrated parasite management practices are recommended.

## Graphical abstract

This study evaluated the efficacy of widely used commercial anthelmintics and examined whether combining different drug classes enhances their effectiveness in naturally infected sheep in the Kaffa Zone. This study suggest the presence of emerging anthelmintic resistance to albendazole and tetramisole in the study area and highlight the potential usefulness of combination anthelmintic strategies.

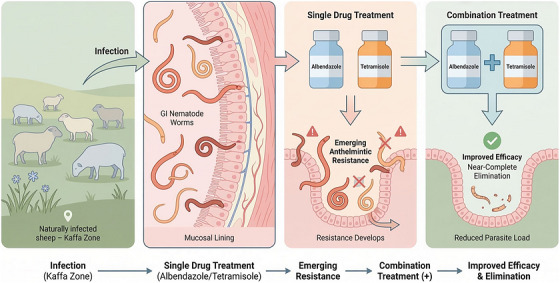

## 1 Introduction

Sheep and goats play a central role in rural households’ livelihood across Ethiopia, contributing to food security, income and the national livestock sector (Bekele et al. 2011). The country possesses one of Africa's largest small ruminant populations, and these animals support both domestic consumption and the export of meat skins and live animals (Kassaye and Kebede 2010, Sheferaw et al. 2010). In spite of this huge potential, the productivity of small ruminants remains below expectations due to persistent challenges such as infectious diseases, low genetic performance, nutritional limitations and traditional management systems (Yami and Merkel 2008).

Among significant health constraints, infections caused by gastrointestinal nematode (GIN) are especially detrimental. These parasites are widely distributed across the country and contribute significantly to economic losses due to decreased weight gain, impaired reproductive performance and heightened vulnerability to other diseases (Fikru et al. 2006). For several years, the management of GIN infections has primarily relied on chemotherapeutic control through the use of broad‐spectrum anthelmintics. While these drugs initially provided reliable control, the effectiveness of several anthelmintic classes has diminished in many regions due to the emergence and spread of drug‐resistant nematode populations (Kumsa and Wosene 2006). Resistance has been linked to factors such as frequent and indiscriminate treatment, reliance on anthelmintics with similar modes of action, and the absence of coordinated parasite‐management programs, all of which have facilitated the selection of resistant nematode populations (Kaplan 2004).

In Ethiopia, the use of anthelmintics such as albendazole, ivermectin and tetramisole has been prevalent in both public and private veterinary services for several decades. These drugs are easily accessible in rural markets and are routinely administered to livestock, accounting for a significant portion of the expenses incurred by the country in the management of helminthosis (Achenef et al. 2013; Desie et al. 2013; Getachew et al. 2013; Takele et al. 2013; Ayalew et al. 2014; Biffa et al. 2006). Despite the continued accessibility of these drugs, reports from different agro‐ecological settings indicate an increasing prevalence of benzimidazole‐ and imidazothiazole‐resistant parasites, raising concerns regarding the sustainability of current parasite‐control strategies. This emerging trend poses a threat to the sustainability of parasite control in small ruminants and underscores the necessity of routine surveillance of drug efficacy (Desie et al. 2013; Takele et al. 2013; Getachew et al. 2016; Haile‐Mariam 2015; Solomon et al. 2024).

In recent years, a strategy that has gained traction to address the declining efficacy of anthelmintics is the implementation of combination anthelmintics therapy (Coles and Roush 1992; Barnes et al. 1995; Leathwick et al. 2009; Bartram et al. 2012). This approach involves the administration of two active compounds that function through distinct mechanisms of action, with the objective of enhancing treatment efficacy and mitigating the emergence of resistant nematode genotypes (Leathwick et al. 2009). By exerting selective pressure on multiple physiological pathways concurrently, these combinations can enhance treatment outcomes and decelerate the development of resistance. The potential advantages of this strategy are particularly relevant in production systems where farmers frequently rely on a limited number of drug classes (Bartram et al. 2012).

In Kaffa Zone, a community‐based gastrointestinal (GIT) parasite control program was introduced in 2018, using anthelmintics such as albendazole and ivermectin with notable reductions in parasite load. Intervention significantly reduced GIT parasite load in sheep with an efficacy of 89.2% against nematodes and 100% against fascioliasis. However, suboptimal faecal egg count reductions observed in some animals, together with farmers’ concerns about declining treatment success, suggested that resistance may be emerging. Until now, no formal evaluation of drug performance has been undertaken to confirm these concerns.

Given these concerns, this study was designed to evaluate the efficacy of commonly used anthelmintics, assess the presence of anthelmintic resistance and determine the effectiveness of combination therapies.

## 2 Materials and Methods

### 2.1 Study Area and Study Population

**FIGURE 1:**
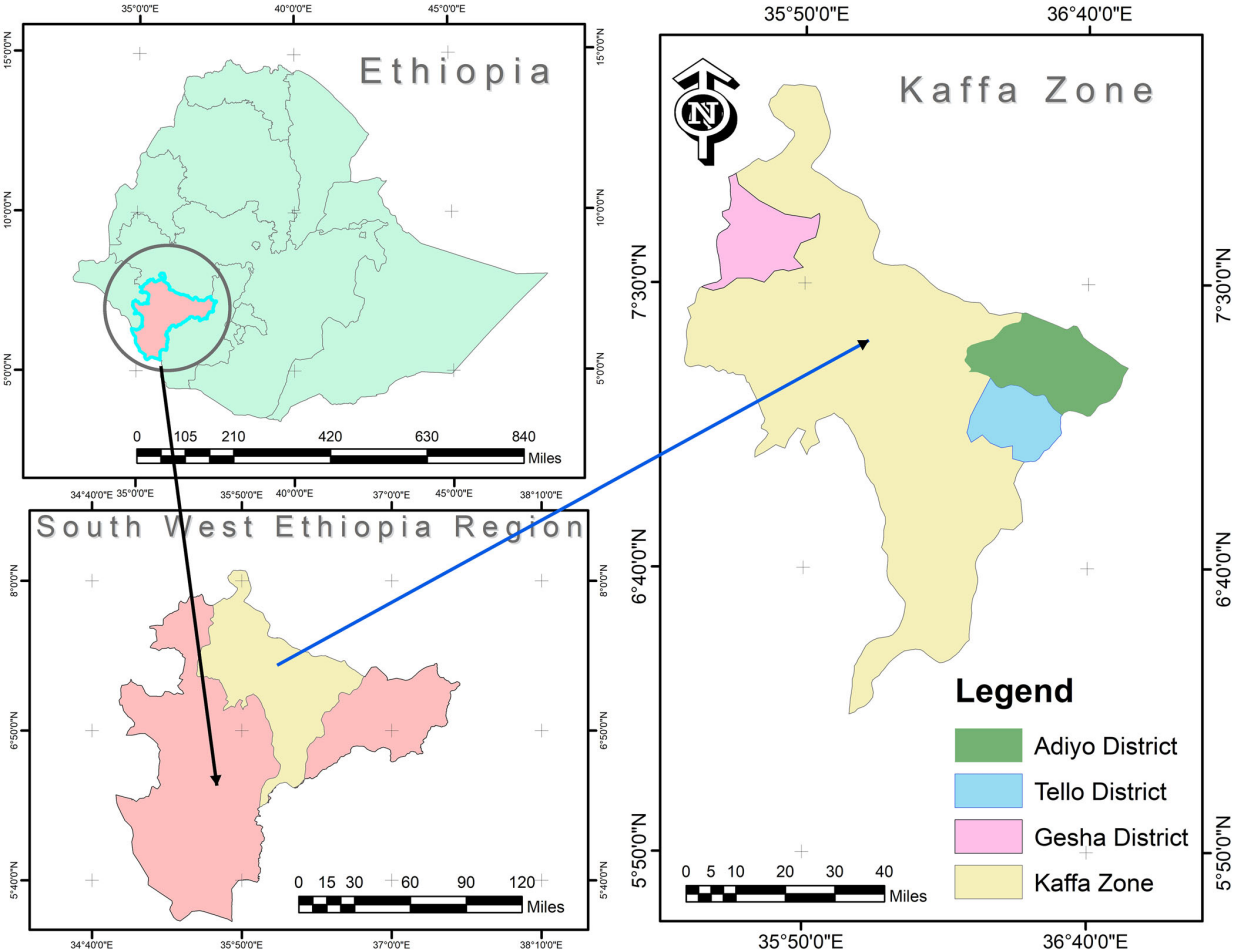
Map showing areas where the study was conducted.

The investigation was carried out in Kaffa Zone of Southwestern Ethiopia, focusing on the districts of Adiyo, Tello and Gesha Districts. This region is situated between 6°24′ and 8°13′ N latitude and 35°30′ to 36°46′ E longitude. The Kaffa Zone is distinguished by its humid highland climate, which receives an estimated annual rainfall of approximately 1700 mm, alongside a mean annual temperature of around 19°C. The geographical landscape consists of mountainous terrain, a dissected plateau, and gently undulating hills, with elevations ranging from 400 to 3100 m above sea level. Livestock production in Kaffa Zone is predominantly based on a mixed crop‐livestock production system mainly characterized by smallholder, subsistence‐oriented and extensive management practices. The major livestock production include cattle rearing and Bonga sheep rearing, which primarily depends on natural grazing lands and crop residues as feed resources (Figure 1).

A randomized control field trial was conducted from September 2024 to January 2025. A total of 275 sheep from community‐based breeding‐program villages, all older than 6 months, were selected for inclusion. Both sexes were represented and only those animals shedding a minimum of 150 eggs per gram (EPG) of faeces were enrolled. Animals with low pre‐treatment egg counts typically have a total count of less than 150 EPG and those animals which received or have been exposed to other anthelmintic or parasiticidal treatments within the previous 4–8 weeks were excluded from the study. Eligible animals were then randomly assigned to different treatment and control groups.

### 2.2 Anthelmintic Identification

A brief questionnaire‐based survey was conducted to gather information on the types of anthelmintic products commonly sold in local veterinary drug shops. Data collected included common anthelmintic drugs available in the local market, their brand, country of manufacture, importation channels and the usage trend of anthelmintic among farmers (Table 1).

**TABLE 1:** Anthelmintics used in the field efficacy evaluation.

Trade name	Generic name	Family name	Manufacturer	Dosage Kg/BW	Route of administration
Ashialben 300	Albendazole	Benzimidazole	Ashish Life Science Pvt. Ltd., India	7.5 mg	Oral
Tetracozash 900	Tetramisole HCl + oxyclozanide	Imidazothiazoles	Ashish Life Science Pvt. Ltd., India	30 mg	Oral
Imectin 1%	Ivermectin (1%)	Macrocyclic lactones	Hebei Kexing Pharmaceutical Co. Ltd., China	0.2 mg	SC injection

### 2.3 Anthelmintics Selected for Experimental Evaluation

Three families of anthelmintics with their respective modes of action and their combination were selected for the experiment. The anthelmintics used include albendazole (benzimidazole group), ivermectin (macrocyclic lactones group) and tetramisole (imidazothiazole group). Also, the combinations of anthelmintics used in the study are albendazole + ivermectin and tetramisole + ivermectin.

### 2.4 Quantitative Faecal Examination

Faecal samples, approximately 3 g each, were procured from every identified positive animal and were subsequently homogenised in 42 mL of a saturated sodium chloride flotation solution. The resultant mixture was filtered to remove coarse materials and transferred into tubes for a brief settling period. Following this, the supernatant from each sample was then loaded into both chambers of a McMaster slide, where nematode eggs within the designated grids were enumerated (Taylor et al. 2007; Soulsby 1986). Egg counts were then converted to EPG and categorised according to established guidelines by Hansen and Perry (1994) as light (up to 800 EPG), moderate (800–1200 EPG) and heavy (> 1200 EPG).

### 2.5 Field Clinical Trial

A field clinical trial was conducted to assess the development of anthelmintic resistance in GIT parasites affecting naturally infected sheep within the designated study area. The study employed an experimental study design featuring a controlled field clinical trial aimed at evaluating the efficacy of anthelmintic treatment. Sheep selected for this study were part of a community‐based breeding program, ensuring a robust assessment of the anthelmintic efficacy test.

#### 2.5.1 Sampling Method and Treatment

Study districts were selected purposively based on the presence of community‐based sheep breading programs and population status of sheep. Within each district, kebeles were selected randomly. All animals were randomly divided into six groups at each kebeles (Group I–VI), with each treatment group containing equal number of animals. Each treatment group had 45 animals and 50 animals were included in the control group. Grouping was based on faecal egg count at Day 1 (before treatment) to minimize variability among groups. Animals in Group I were kept as controls without any treatment. Groups II, III, IV, V and VI were administered with albendazole, tetramisole, ivermectin, albendazole + ivermectin and tetramisole + ivermectin, respectively, according to the recommended route and dose of the manufacturer. Before starting treatment, all animals were identified by ear tagging and necessary measurements. Faecal egg counts were carried out by the McMaster counting method (Soulsby 1986).

#### 2.5.2 Evaluation of Anthelmintic Efficacy and Resistance

Anthelmintic efficacy was evaluated using the faecal egg count reduction test (FECRT) as recommended by Coles et al. (1992). Faecal samples were collected on the day of the treatment and again 10 days later for albendazole and tetramisole and 14 days later for ivermectin and combination treatments (Geary et al. 2012). Arithmetic mean egg counts were calculated for both treated and untreated control groups. The percentage reduction in egg counts was derived using the standard formula:

$$\begin{array}{ccc} \mathrm{FECR}\% & = & \left[1-\left(\mathrm{arithmetic}\;\mathrm{mean}\;\mathrm{of}\;\mathrm{post}\right.\right.\\ & & \;\;-\;\mathrm{treatment}\;\mathrm{EPG}\;\mathrm{of}\;\mathrm{the}\;\mathrm{treated}\;\mathrm{group}\left(X_{t}\right)\;/\\ & & \;\;\mathrm{arithmetic}\;\mathrm{mean}\;\mathrm{of}\;\mathrm{post}-\mathrm{treatment}\;\mathrm{EPG}\\ & & \;\left.\mathrm{of}\;\mathrm{the}\;\mathrm{control}\;\mathrm{group}\left(X_{c}\right)\right]\;\times \;100 \end{array}$$

A reduction of FECRT% below 95% and a lower 95% CI of less than 90% was interpreted as suggestive of reduced efficacy or potential resistance (Coles et al. 1992).

### 2.6 Data Management and Analysis

The data collected during sampling and laboratory findings were entered and stored into an MS‐Excel spreadsheet. Before being subjected to statistical analysis, the data were thoroughly screened for errors and were properly coded. Stata version 14.2 software package was used to perform the statistical analysis. Descriptive statistics were used to represent the results for the anthelmintic efficacy of the drugs. In all analyses, confidence level was held at 95% and *p *< 0.05 was set for significance value. The anthelmintic efficacy test was evaluated by calculating FECRT% by applying the formula *E* = [1 − (*T*/*C*)] × 100, where *E* = percentage efficacy; *C* = mean number of eggs in the control group and *T* = mean number of eggs in the treated group; where the value of FECRT% less than 95% will be suspected as developed resistance or poor efficacy (Coles et al. 1992).

## 3 Results

The pre‐treatment assessment of the proportion of the level of intensity of GIN infection of sheep used in the study revealed that heavy infection (69.8%) was predominant in the study area (Table 2). This might be due to the high parasitic rainy season when the study was conducted, environmental factors, and management factors. The range of faecal egg counts of infected sheep was from 350 to 6750 EPG before treatment. The overall mean of faecal egg counts of infected sheep pre‐treatment was 1881.5 ± 69.7 (95% CI: 1744.2–2018.9). The range of faecal egg counts of infected sheep was from 0 to 1550 EPG after treatment. The overall mean of faecal egg counts of infected sheep post‐treatment was 229.9 ± 28.4 (95% CI: 174.1–285.8) (Table 3).

The FECRT result revealed values 90.31% (lower 95% CI: 88.51%), 91.39% (lower 95% CI: 89.53%) and 95.84% (lower 95% CI: 95.05) for albendazole, tetramisole and ivermectin, respectively (Table 4).

**TABLE 2:** Pre‐treatment distribution of gastrointestinal nematode infection intensity in sheep.

Level of Intensity	No of Positive	Mean EPG ± SE	Proportion (%)
Light	15	556.7 ± 39.3	5.5
Moderate	68	1002.2 ± 13.7	24.7
Heavy	192	2295.6 ± 74.2	69.8

The two anthelmintic combinations—albendazole with ivermectin and tetramisole with ivermectin showed markedly higher reductions in egg counts compared to the single active formulations. FECRT values were 99.11% and 99.27%, respectively, with corresponding confidence limits well above 97%, demonstrating strong performance against GINs.

When we see the suspected resistance of selected anthelmintics based on study areas, resistance was suspected for albendazole and tetramisole at Adiyo district based on the FECR% (90.68% and 90.97%). Similarly, resistance was suspected for albendazole and tetramisole in Gesha district (90.57% and 90.98%). On the contrary, in Tello district, resistance was suspected for albendazole and ivermectin. Resistance was not suspected for tetramisole at Tello district, and the effectiveness of tetramisole might be attributed to less frequent use of the drug for deworming in the study area (Table 5 ).

**TABLE 3:** Mean of EPG pre and post‐treatment.

Parameter	Pre‐treatment	Post‐treatment
Max	6750	1550
Min	350	0
Mean EPG ± SE	1881.5 ± 69.7	229.9 ± 28.4
95% CI	1744.2–2018.9	174.1–285.8

**TABLE 4:** Mean faecal egg counts before and after treatment, and FECR% for different anthelmintic groups.

	Mean FEC ± SD			95% CI	
Anthelmintic	Pre‐treatment	Post‐treatment	FECR%	Lower	Upper
Albendazole	1784.91 ± 142.7	190.56 ± 23.1	90.31	88.51	92.1
Tetramisole	1930.56 ± 167.43	176.85 ± 25.07	91.39	89.53	93.25
Ivermectin	1972.64 ± 125.81	81.13 ± 7.76	95.84	95.05	96.63
Albendazole + Ivermectin	2001.43 ± 227.42	51.43 ± 9.28	99.11	97.07	99.55
Tetramisole + Ivermectin	1985.29 ± 189.03	38.24 ± 5.61	99.27	97.75	99.8
Control	1377.5 ± 174.96	1510 ± 158.68	—	—	—

**TABLE 5:** Mean faecal egg counts and FECR% after treatment in the three study sites.

	Treatment								
	Albendazole			Tetramisole			Ivermectin		
Study Site	Pre‐treatment	Post‐treatment	FECR%	Pre‐treatment	Post‐treatment	FECR%	Pre‐treatment	Post‐treatment	FECR%
Adiyo	1710 ± 183.13	176.67 ± 27.2	90.68	1660 ± 245.14	161.67 ± 34.46	90.97	2013.3 ± 148.8	76.6 ± 9.5	96.37
Gesha	1850 ± 249.24	195 ± 44.41	90.57	2115 ± 204.69	215 ± 42.94	90.98	1965 ± 249.98	82.5 ± 13.71	95.43
Tello	2100 ± 680.69	300 ± 76.38	84.9	3037 ± 594.9	100 ± 20.41	96.62	1616.7 ± 169.1	116.7 ± 44.1	93.17

## 4 Discussion

GIN infections remain widespread in Ethiopia and pose a significant challenge to small ruminant production. Their control efforts have long depended on repeated use of broad‐spectrum anthelmintic. This practice has unfortunately facilitated the gradual emergence of drug‐resistant populations of parasites (Fikru et al. 2006). Several factors including incorrect dosing, frequent whole‐flock treatments, unregulated drug availability and inconsistent treatment schedules are commonly cited as contributors to the development and spread of resistance (Kassahun et al. 2016).

In the current study, the recorded results revealed FECRT percentage of 90.31%, 91.39% and 95.84%, along with corresponding lower 95% confidence limit of 88.51, 89.53, and 95.05 for albendazole, tetramisole and ivermectin, respectively. These results provide an evidence of a concerning reduction in the efficacy of both albendazole and tetramisole in the study area, as reflected by FECRT values below the established 95% threshold and lower confidence limits below 90%. Prolonged dependence on these drug classes, coupled with frequent blanket treatments and inconsistent dosing practices, has likely played a significant role in the selection and proliferation of resistant nematode populations in the region (Leignel et al. 2010; Furtado et al. 2016).

The diminished effectiveness of albendazole noted in this study concurs with multiple prior observations from Ethiopia and elsewhere. This is consistent with the reports of Getachew et al. (2016) who reported 57.5% FECR% at Areka Agriculture Research Centre, as well as to the report of Solomon et al. (2024), who reported 90.4% FECR% in Nejo district, Oromia, Ethiopia. Similarly, Mondragón‐Ancelmo et al. (2019) indicated an FECR% of 83% with a 95% lower confidence limit of 75% for albendazole. Conversely, these findings disagree with the studies conducted by Sibhatu et al. (2011), Achenef et al. (2013), Terefe et al. (2013), Bahiru et al. (2017) and Seyoum et al. (2017), all of which reported the effectiveness of albendazole against of nematode parasites with FECR% exceeding 96%.

Tetramisole also showed indication of reduced performance, a conclusion that aligns with the findings of Asmare et al. (2005) from Southern Ethiopia, who documented a 97.5% FECR with 85% lower limit of the 95% confidence interval. Similarly, Melaku et al. (2013) from Northwest Ethiopia reported an FECR of 84.87% FECR with a lower limit of 73.95% for the 95% confidence interval when using tetramisole. In contrast, reports of various studies in Ethiopia by Kumsa and Nurfeta (2008), Sibhatu et al. (2011), Getachew et al. (2016) and Solomon et al. (2024) have reported higher efficacy against nematodes. Variation in the degree of resistance across regions likely reflects differences in treatment frequency, parasite populations and product quality, suggesting that resistance remains unevenly distributed (Kumsa et al. 2010).

The findings regarding the effectiveness of ivermectin against GINs in the present study align with numerous investigations conducted on nematodes of sheep maintained under the extensive system by smallholder farmers in Ethiopia (Desie et al. 2013; Seyoum et al. 2017; Asmare et al. 2005; Kumsa and Nurfeta 2008; Menkir et al. 2006; Chaka et al. 2009). This correlation suggests that macrocyclic lactones maintain robust activity against local GIN populations. Nevertheless, the results accrued in the current study diverge from prior research conducted in other areas by Kumsa and Abebe (2009), Getachew et al. (2016) and Solomon et al. (2024). This highlights the importance of employing rational and judicious use of ivermectin to safeguard its long‐term effectiveness.

Chemotherapeutic medications with similar spectra and different modes of action have a well‐established history of combination use in the treatment of the most alarming diseases, including cancer (Humphrey et al. 2011), bacterial diseases (Fischbach 2011) and viral infections (Ryom et al. 2018). This strategic use of drug combination has been fruitful in accomplishing improved efficacy, decreased toxicity and reduced development of drug resistance. In ruminants, anthelmintic drug combinations with nematodicidal activity can be employed to postpone resistance development, to manage prevailing field resistance and/or target specific species that limit effective dosing (Bartram et al. 2012; Geary et al. 2012). Numerous studies have emphasized that combination therapy yields optimal outcomes when introduced prior to the widespread emergence of resistance among all component drugs. In these scenarios, such combinations act as a protective strategy, prolonging the effectiveness of current anthelmintic classes (Bartram et al. 2012; Dobson et al. 2011; Leathwick et al. 2012; Leathwick et al. 2015; Canton et al. 2017; Lanusse et al. 2018).

Based on the current study's result, when used in combination, albendazole with ivermectin, as well as tetramisole with ivermectin combinations consistently outperformed the individual drugs, achieving a faecal egg count reduction percentage (FECR%) of 99.11% and 99.27%, respectively. This improved efficacy of these combinations added to the body of evidence indicating that multi‐active formulations offer a valuable safeguard in regions where resistance to one or more single‐active ingredients is emerging (Leathwick et al. 2012; Canton et al. 2017; Bartley et al. 2004; Bartley et al. 2005; Entrocasso et al. 2008; Le Jambre et al. 2010). By acting on different biological targets, combination treatments reduce the likelihood that resistant parasites surviving treatment, thereby helping to impede the proliferation of resistant genotypes within the population (Bartram et al. 2012; Van Wyk 2001).

## 5 Conclusion

The present study has highlighted that both albendazole and tetramisole showed reduced efficacy in providing optimal control of GINs in sheep within the Kaffa zone, as evidenced by reduced faecal egg count reduction values. The FECRT yielded a percentage of 90.31% and a lower 95% confidence limit of 88.51 for albendazole, and 91.39% and 89.53 for tetramisole, in contrast to ivermectin, which demonstrated a reduction percentage of 95.84% with a lower confidence limit of 95.05%. While ivermectin maintained acceptable levels of activity, continued vigilance is necessary given the growing reports of reduced ivermectin sensitivity elsewhere. Moreover, the result for the combination of anthelmintics—albendazole with ivermectin and tetramisole with ivermectin—showed faecal egg count reduction percentage of 99.11 and 99.27 FECR%, respectively, indicating that multi‐active treatment strategies may offer a practical means of improving parasite control in areas experiencing resistance to single‐agent drugs. Overall, the results reaffirm the necessity of routine monitoring of anthelmintic performance and indicate that adopting combination therapies could reinforce parasite control programmes in areas facing the challenge of developing resistance to conventional drugs. Therefore, to ensure long‐term sustainability, it is recommended that such pharmacological strategies should be complemented by broader management approaches, including targeted treatment, improved grazing management and regular assessment of parasite burdens.

## Author Contributions

**Nahom Belay**: conceptualization, investigation, writing – original draft, methodology, writing – review and editing, visualization, validation, software, supervision. **Asrat Arke**: investigation, writing – review and editing, methodology, data curation, validation, visualization, formal analysis**. Lamrot Tesfaye**: investigation, writing – review and editing, visualization, methodology, data curation.

## Funding

The authors have nothing to report.

## Ethics Statement

The authors confirm that all ethical policies outlined in the journal's author guidelines have been fully adhered to and approval was obtained from the Animal Research Ethics Committee of Bonga Agricultural Research Center (Approval Number: BARC/AREC/011/2017/24). All procedures were conducted in accordance with standard veterinary parasitological guidelines and animal welfare principles.

## Conflicts of Interest

The authors declare no conflicts of interest.

## Data Availability

The data used in this study are available from the corresponding author upon reasonable request.
